# Prevalence and Correlates of Problem Gambling in a Representative Sample of Norwegian 17-Year-Olds

**DOI:** 10.1007/s10899-014-9455-4

**Published:** 2014-03-12

**Authors:** Daniel Hanss, Rune A. Mentzoni, Alex Blaszczynski, Helge Molde, Torbjørn Torsheim, Ståle Pallesen

**Affiliations:** 1 Department of Psychosocial Science, Faculty of Psychology, University of Bergen, Post box 7807, 5020 Bergen, Norway; 2 School of Psychology, The University of Sydney, Sydney, Australia; 3 Department of Clinical Psychology, Faculty of Psychology, University of Bergen, Bergen, Norway

**Keywords:** Adolescent gambling, Prevalence, Risk factors, Gambling attitudes, PGSI, Norway

## Abstract

We report data collected in a representative sample of 17-year-old Norwegians to investigate prevalence rates of non-problem, risk, and problem gambling, as measured by the Problem Gambling Severity Index (PGSI). In addition, we explored the importance of demographic, personality, motivational, social, and health variables explaining variance in adolescent gambling. Prevalence rates of risk and problem gambling were low but similar to those found in previous studies outside of Norway using the PGSI in adolescent samples. With regard to the relative importance of the various covariates, we found that motivational variables (future gambling intentions, attitudes toward gambling, and gambling-related knowledge) distinguished best between those who did not gamble, non-problem gamblers, and risk and problem gamblers. Furthermore, social variables were important covariates of adolescent gambling; significant associations were found for family and friends’ approval of gambling, parental monitoring, father’s level of education, and having relatives or friends with a history of a gambling disorder. We discuss possible reasons for differences between the covariates with regard to their importance for explaining adolescent gambling and address implications for future research.

## Introduction

Adolescents are exposed to many opportunities to gamble, for example, scratchcards received as birthday presents, participation in online casinos, and poker games with friends. Despite underage gambling being illegal—including the distribution of lottery tickets to youth—research from several countries, among them Norway, indicates that many adolescents are active gamblers (Cronce et al. 2007; Wood and Griffiths 2004; Derevensky et al. 2010). For example, in a recent survey among high school students in Australia 56 % of participants answered that they had gambled in the previous 12 months (Delfabbro et al. 2009). Thirty percent of the participants of a Swedish national representative survey answered that they had gambled before turning 18 years old (Statens folkhälsoinstitut 2010).

The majority of adolescents who gamble appear not to experience serious negative consequences. In fact, adolescents’ reasons for engaging in gambling include many positive aspects such as enjoyment, excitement, relaxation, and social involvement (Gupta and Derevensky 1998). However, some adolescent gamblers report the presence of serious gambling problems, including difficulties controlling their gambling, gambling-related conflicts with family and friends, and financial problems due to losses (Wiebe et al. 2000). A meta-analysis of studies that investigated gambling activities of college students in the USA and Canada revealed that between 5.4 and 10.4 % (95 % confidence interval) of students reported serious gambling-related problems (Blinn-Pike et al. 2007). More recently, an international review showed that prevalence studies conducted from 1997 to 2007 among adolescents in Nordic countries (Denmark, Finland, Iceland, Norway, and Sweden) classified between 0.8 and 3.2 % of youth as problem/pathological gamblers (Volberg et al. 2010).

Why is gambling an enjoyable and unproblematic recreational activity for some adolescents, but others experience serious gambling-related problems, and yet others do not gamble at all? Research can shed light on this question by identifying variables that distinguish between these different groups of adolescents. A significant body of literature is devoted to this topic and factors that have been found to be associated with adolescent problem gambling include male gender, personality (e.g., impulsivity, sensation seeking), attitudes toward gambling (e.g., believing that gambling has potential benefits for individuals and society), family gambling history and approval of gambling, and mental health (for a more comprehensive overview of adolescent gambling covariates see Derevensky and Gupta 2004; Hardoon and Derevensky 2002). One of the limitations of the existing literature is that most studies have focused on a limited range of covariates, and, therefore, little is known about the relative importance of a wider range of covariates in explaining adolescent gambling (Langhinrichsen-Rohling et al. 2004).

In this study, we report prevalence rates of problem gambling among a representative sample of Norwegian 17-year-olds, and the extent to which demographic, personality, motivational, social, and health variables were associated with gambling and problem gambling in this age group. The groups of variables (e.g., motivational variables) were compared with regard to their associations with gambling and problem gambling, and within each group of variables we explored the relative importance of single covariates (e.g., attitudes toward gambling, future gambling intentions). We used the label *problem gambling* to remain consistent with the measurement instrument employed in this study, although the term used to describe problem and pathological gambling was replaced by *gambling disorder* in the Fifth edition of the Diagnostic and Statistical Manual of Mental Disorders (American Psychiatric Association 2013).

Before we turn to our study, we will provide a selective overview of previous research. The studies included in our overview used different measures, classifications, and terms to describe subgroups of gamblers (for a discussion of this issue see Blaszczynski et al. 2004).

### Demographic Variables Associated with Problem Gambling

Previous research among adolescents has demonstrated that male gender (Chalmers and Willoughby 2006; Dickson et al. 2008; Felsher et al. 2003; Nower et al. 2004; Wickwire et al. 2007; Scott Brunborg et al. 2013; Molde et al. 2009) and membership of an ethnic minority group (e.g., Langhinrichsen-Rohling et al. 2004) are risk factors for problem gambling. In addition, it has been shown that those who had been raised by both parents were less likely to be classified as a probable pathological gambler compared to those who had been raised by one parent only or in another family constellation (Langhinrichsen-Rohling et al. 2004).

### Personality Variables Associated with Problem Gambling

Among the personality variables that have received most attention in connection with adolescent gambling are the Big five personality domain traits Neuroticism, Extraversion, Openness, Agreeableness, and Conscientiousness (Costa and McCrae 1992; McCrae and Costa 2003), impulsiveness (Eysenck and Eysenck 1977), and sensation seeking (Arnett 1994). In one study, it was found that Neuroticism, Openness, impulsiveness, and the sensation seeking sub-dimension Intensity (Arnett 1994) distinguished between pathological and non-pathological gamblers (Myrseth et al. 2009). Pathological gamblers scored higher on Neuroticism, impulsiveness, and Intensity but lower on Openness than non-pathological gamblers. In other studies, Neuroticism was positively related to problem gambling (Bagby et al. 2007; Kaare et al. 2009) with Conscientiousness found to be negatively related to the condition. Clarke (2006) showed that impulsiveness was positively associated with gambling problems among adult university students, which has also been shown among adolescents (Vitaro et al. 2004; Nower et al. 2004). With regard to the relation between sensation seeking and problem gambling, an earlier review showed that the findings are contradictory (Hammelstein 2004). While a recent meta-analysis concluded that sensation seeking is not elevated in problem gamblers as compared to non-problem gamblers (MacLaren et al. 2011), three studies among adolescents suggest that sensation seeking is associated with gambling problems (Nower et al. 2004; Gupta et al. 2006; Wanner et al. 2006).

### Motivational Variables Associated with Problem Gambling

Motivational variables that have received attention in the adolescent gambling literature include attitudes toward gambling, gambling-related knowledge, and future gambling intentions. Several studies have shown that positive attitudes toward gambling activities are related to gambling frequency (Wood and Griffiths 2004) and risk and problem gambling (Delfabbro and Thrupp 2003; Delfabbro et al. 2009; Williams et al. 2006). With regard to gambling-related knowledge, findings indicate that regular gamblers tend to hold erroneous beliefs, including misconceptions of control over and randomness of outcomes in gambling; for an overview see Hardoon and Derevensky (2002). It has been argued that such misconceptions combine with hope for monetary gains in motivating people to gamble. Gambling-related misconceptions have also been demonstrated in adolescent samples. Delfabbro et al. (2009) found that adolescent problem gamblers were more likely than at-risk and not-at-risk gamblers to think that gambling involves a skill component. Those with gambling problems were more likely than at-risk and not-at-risk gamblers to misunderstand randomness in coin toss games and playing poker. However, the same study also found that adolescents with gambling problems had *better* understanding than not-at-risk gamblers of the objective probabilities involved in roulette. No group differences were found with regard to participants’ understanding of the probabilities involved in a coin toss game and a lottery. Findings are also mixed with regard to future gambling intentions. An early study (Moore and Ohtsuka 1997) found that intentions to gamble in the future were positively associated with gambling frequency and gambling problems among 14–25 year-olds. However, a more recent study indicated that gambling intentions of adolescents who engaged in risk and problem gambling did not differ from those who did not engage in risk and problem gambling (Delfabbro and Thrupp 2003).

### Social Variables Associated with Problem Gambling

Among the social variables that have been investigated in connection with adolescent gambling are family and peer gambling history and approval of gambling, parental monitoring, family cohesion, and parents’ level of education. Common findings are that higher levels of parental gambling (Felsher et al. 2003; Magoon and Ingersoll 2006) and having family members with gambling problems (Cronce et al. 2007; Delfabbro and Thrupp 2003; Dickson et al. 2008) are associated with risk and problem gambling. Vachon et al. (2004) found that the severity of problem gambling on the father’s but not the mother’s side was associated with the severity of problem gambling among adolescents. How often adolescents gambled was, however, associated with gambling frequency and problem gambling on both the father’s and mother’s side. The relationship between parental gambling history and offspring gambling problems was confirmed in a longitudinal study (Winters et al. 2002). In terms of parental monitoring it has been demonstrated that this may be a protective factor for gambling problems among adolescents (Magoon and Ingersoll 2006; Wanner et al. 2006), particularly for girls (Chalmers and Willoughby 2006). With regard to family cohesion the findings are mixed. Dickson et al. (2008) showed a negative association between family cohesion and gambling severity among adolescents. Another study found no relationship between these variables (Langhinrichsen-Rohling et al. 2004). The relation between parents’ level of education and adolescent gambling seems to be weak at best. Mother’s level of education was weakly associated with frequency of gambling among female (inverse relation) but not male adolescents (Barnes et al. 2005). Several other studies found that parents’ education was unrelated to gambling frequency (Floros et al. 2013; Martins et al. 2008) and level of gambling severity among adolescents (Langhinrichsen-Rohling et al. 2004; Chalmers and Willoughby 2006). In addition, attitudes and behaviors of peers may also be influential. Having peers who approve of gambling and who gamble has been shown to be positively associated with levels of gambling frequency and problem gambling (Magoon and Ingersoll 2006; Dickson et al. 2008; Delfabbro and Thrupp 2003).

### Health Variables Associated with Problem Gambling

It has been hypothesized that people with gambling problems more often than recreational gamblers engage in gambling in order to escape dysphoric feelings such as depression (Gupta and Derevensky 1998; Clarke 2006), loneliness (Porter et al. 2004), low personal well-being (Chalmers and Willoughby 2006), and anxiety (Ste-marie et al. 2002, 2006; Wanner et al. 2006). Some evidence exists to suggest that the relationship between gambling and depression is mediated by impulsiveness (Clarke 2006).

### Research Aims

This study aimed to determine prevalence levels of non-problem, risk, and problem gambling in a representative sample of Norwegian 17-year-olds. A further aim was to explore how well the various variables reviewed above would distinguish between non-gamblers, non-problem gamblers, and risk- and problem gamblers.

Most previous studies on adolescent gambling have focused on *one* of the above groups of variables, and, hence, little is known about the relative importance of the variables for explaining adolescent gambling. Therefore, in this study, demographic, personality, motivational, social, and health variables were all included to compare their associations with adolescent gambling at two levels: (a) a comparative analysis of the different groups of variables (e.g., how important are motivational compared to social variables?) and (b) a similar analysis of the single variables within one group of variables (e.g., for the motivational variables: how important are attitudes relative to intentions?). In addition, the study integrated all of the variables to explore how well they explain problem gambling together. Given the study was exploratory, no specific hypotheses were formulated regarding the associations between the variables and the relative importance of the independent variables for explaining adolescent gambling.

## Method

### Participants and Procedure

A sample of 3,000 17-year-olds (*n* = 1,500 female) was randomly drawn from the Norwegian National Registry and informed about the study by means of a pre-notification letter. The letter described the purpose of the study (i.e., determining to what degree 17-year-old Norwegians are involved in gambling activities), the researchers' affiliation (University of Bergen), and that all participants would receive a gift card worth NOK 200 (approximately € 27) as a compensation for taking part in the study.

The actual questionnaire was sent one week after the pre-notification letter had been sent. It included an informed consent letter and a pre-paid return envelope. Those who decided to participate could choose between completing the paper version of the questionnaire or, alternatively, an online version of the questionnaire. The link to the online questionnaire was provided in the informed consent letter. Up to two reminder letters were sent to those who did not respond to the first questionnaire sending.

Of the 3,000 individuals contacted 2,059 completed the questionnaire.^1^ Of these, four participants had to be excluded because they were younger than age 17. Just over half of the remaining 2,055 participants were female (52.9 %, *n* = 1,088). The majority of those who participated lived together with both of their parents (61.9 %, *n* = 1,271) and were full-time students (e.g., high school, vocational school; 97.7 %, *n* = 2,007). The mean school grade point average was *M* = 4.16, *SD* = 0.72 (grade range is 1-6, where 6 is the best). Only a few participants worked full-time (*n* = 10) or were on sick leave (*n* = 4); a larger proportion worked part-time (20.1 %, *n* = 412). The average number of siblings was *M* = 2.11 (*SD* = 1.38).

The response rate was 70.4 % (i.e., 2,059 of 2,923) after excluding 54 people with invalid addresses (could not be reached) and 23 whose parents informed us that their child was not able to take part in the study (e.g., due to studies abroad, disability etc.).

### Measures

#### Gambling Problems

The Problem Gambling Severity Index (PGSI) included in the Canadian Problem Gambling Index (CPGI) (Ferris and Wynne 2001) was used to assess gambling status. Although the PGSI was originally developed for use in the general adult population, recent studies have used the instrument in samples including adolescents (Statens folkhälsoinstitut 2010) and young adults (Delfabbro et al. 2013). Because the participants of this study will be followed up at ages 18 and 19 it was important that the instrument to measure gambling problems could be used for both adolescents and adults.

Five of the nine PGSI items address problematic gambling behavior and the remaining four items address negative consequences of gambling. The participants answered the items on a four-point rating scale ranging from *never* (0) to *always* (3). Cronbach’s alpha across the nine PGSI items was *α* = .85.

Based upon the individual sum score across the nine items and additional information about the individual gambling behavior during the previous month (described below) each participant was assigned to one of five categories: Non-gambling (*no* gambling during previous month and PGSI sum score of 0), non-problem gambling (gambling during previous month and PGSI sum score of 0), low risk gambling (gambling during previous month and PGSI sum score of 1–2), moderate risk gambling (gambling during previous month and PGSI sum score of 3–7), and problem gambling (gambling during previous month and PGSI sum score of 8 or higher).

#### Gambling Behavior During Previous Month

The participants were presented with 15 gambling types available in Norway (e.g., slot machines, lotto, scratchcards) and asked to indicate (*yes* vs. *no*) whether they had played each of the games during the past month. In addition, participants could state any other, unlisted gambling type that they had engaged in.

Participants were also asked to specify how many days they had participated in gambling (overall, not separately for different types of gambling) and how much money (in NOK) they had lost on gambling during the last month. The latter question did not probe whether any money lost included earlier gains from gambling.

Those who did not select any of the gambling options were assigned to the non-gambling category; exception: a few participants (*n* = 19) did not select any gambling option but answered that they had gambled on several days and lost money on gambling during the previous month—those participants were assigned to one of the gambling categories (i.e., non-problem, low risk, moderate risk, or problem) depending on their PGSI sum score.

#### Demographic Variables

A number of demographic variables were assessed, including the participant’s gender (male vs. female), living situation (living with both parents vs. other), place of birth (Norway vs. other), and ethnicity (primary language spoken at home: Norwegian vs. other). In addition, one item measured school and occupational status (attend school full time, attend school part time, work full time, work part time, sick leave/not able to work, other; more than one option could be chosen).

#### Personality Variables

The five-factors model personality domain traits (Extraversion, Agreeableness, Conscientiousness, Neuroticism, and Intellect/imagination) were measured using the Mini-IPIP scales (Donnellan et al. 2006). The Mini-IPIP scales comprise 20 items; four for each of the five personality traits. Each item consists of a statement and participants are asked to rate how accurately the statement describes them on a five-point scale ranging from *very inaccurate* (1) to *very accurate* (5). For each personality trait, an index variable was computed by averaging participants’ scores on the corresponding items. Cronbach’s alpha values were *α* = .79 (Extraversion), *α* = .71 (Agreeableness), *α* = .66 (Conscientiousness), *α* = .66 (Neuroticism), and *α* = .62 (Intellect/imagination).

Impulsivity was measured by means of the 13-item Eysenck Impulsivity Scale–Narrow Impulsiveness Subscale (Eysenck and Eysenck 1977). Each item consists of a question about specific behaviors (e.g., “Do you often buy things on impulse?”) and the participants are asked to indicate whether they typically act as it is described in the question (i.e., *yes*, scored 1 or *no*, scored 0). We computed an impulsivity index by summing up the scores across the 13 items. The Kuder-Richardson 20 (KR20) reliability coefficient for the 13 items was .74.

Sensation seeking was measured by means of the Arnett Inventory of Sensation Seeking (AISS) (Arnett 1994). The AISS consists of 20 items that cover two dimensions of sensation seeking: Novelty and Intensity (each dimension is measured by 10 items). Each item consists of a statement and the participants are asked to indicate how well the statement describes them. The four-point answer scale ranges from *does not describe me at all* (1) to *describes me very well* (4). Cronbach’s alpha values were *α* = .49 (Novelty) and *α* = .56 (Intensity). Because the internal consistencies of the two subscales were low, a composite sensation seeking index was computed by averaging participants’ scores across the 20 items. Cronbach’s alpha across all 20 items was *α* = .64.

#### Motivational Variables

Attitudes were measured using the Attitudes Towards Gambling Scale (ATGS; Orford et al. 2009). The scale comprises 14 items each consisting of a statement measuring either a positive (e.g., “Gambling is a harmless form of entertainment”) or a negative (e.g., “Gambling is like a drug”) attitude toward gambling. Participants are asked to indicate how much they agree with each statement on a five-point answer scale ranging from *strongly agree* (1) to *strongly disagree* (5). After reverse coding statements that represent positive attitudes a composite ATGS score was computed by adding up participants’ scores on the 14 items. Cronbach’s alpha was *α* = .83.

Three items adopted from Delfabbro and Thrupp (2003) assessed future gambling intentions: “I can’t wait to turn 18 so I can go to adult gambling venues”, “When I turn 18, I will gamble a lot more than I do now”, and “In the future, I will definitely like to gamble regularly.” Participants are asked to indicate how much they agree or disagree with the statement on a five-point scale ranging from *strongly disagree* (1) to *strongly agree* (5). A future gambling intention index was computed by averaging participants’ scores on the three items. Cronbach’s alpha was *α* = .90.

In addition to gambling attitudes and intentions, participants’ perceived level of gambling-related knowledge was measured by two questionnaire items: “I know how most gambling games work” and “I could easily grasp how most gambling games work”. Each item was answered on a five-point scale ranging from *strongly disagree* (1) to *strongly agree* (5). A knowledge index was computed by averaging participants’ scores on the two items. Cronbach’s alpha was *α* = .74.

#### Social Variables

The Parental Monitoring Scale (Silverberg and Small 1991) was used to assess participants’ perceived level of parental monitoring. Each of the six items consists of a statement, for example, “My parents know where I am after school/work”, and the participants are asked to indicate how often the situation described in the item applies. The five-point answer scale ranges from *never* (1) to *always* (5). A composite score was computed by averaging participants’ scores across the six items. Cronbach’s alpha was *α* = .85.

Parents’ level of education was assessed by means of two items, separately for the participants’ mother and father: “What is the highest education of your mother/father?” Answer alternatives were identical for both items: (a) lower secondary, (b) high school, (c) vocational school, and (d) university/college. For each parent a dichotomous variable was computed that indicated whether the parent’s highest education was lower secondary (scored 0) or higher than lower secondary (scored 1).

The six-item Family Relations/Cohesion scale (Oregon Mentors) was employed to assess the perceived level of family cohesion. Participants indicate how well each item describes their own family on a four-point answer scale ranging from *not true* (1) to *always true or almost always* (5). A family cohesion index was computed by averaging participants’ scores on the six items. Cronbach’s alpha was *α* = .84.

Family gambling history was assessed separately for father, mother, and close others (family or friends) (Ellingson et al. 2010). For each of these persons, the participants were to indicate whether or not they had ever (a) gambled in their lifetime, (b) gambled at least once a month for at least 6 months, (c) gambled at least once a week for at least 6 months, and (d) had a period in their life when they had economic, family, legal, work, or emotional problems because of their gambling behavior. For each of the four questions, we calculated the share of persons (i.e., *n* out of 3) to whom the respective statement applied. The first three questions measured non-pathological gambling involvement and the fourth question, pathological gambling.

Four questionnaire items adopted from Delfabbro and Thrupp (2003) were used to assess family and friends’ approval of gambling. An example item is: “Most of my friends gamble a lot”. Participants are to indicate how much they agree with each item on a five-point scale ranging from *strongly disagree* (1) to *strongly agree* (5). An index was computed by averaging participants’ scores on the four items. Cronbach’s alpha was *α* = .75.

#### Health Variables

Anxiety and depression were measured by the Hospital Anxiety and Depression Scale (HADS) (Zigmond and Snaith 1983). The HADS comprises 14 items; seven items measure anxiety, and the other seven items measure depression. Two composite scores, one for anxiety and one for depression, were computed by adding up participants’ scores on the respective items. Cronbach’s alpha values were *α* = .76 (anxiety) and *α* = .69 (depression).

In addition, loneliness was measured by means of an eight-item scale provided by Roberts et al. (1993). Each item consists of a statement, and participants are asked to indicate on a four-point answer scale ranging from *never* (0) to *often* (3) how frequently they feel as it is described in the statement. After reverse-coding four of the items a composite score was computed by adding up participants’ answers on the eight items. Cronbach’s alpha was *α* = .76.

### Data Analysis

Data were analyzed with the statistical package IBM SPSS Statistics 21. Prevalence rates were computed by determining the share of participants belonging to each of five gambling categories (a description of the categories is provided in the Section Gambling Problems above). Chi square and Fisher’s Exact tests were used to compare the gambling behaviors of those who were classified as risk and problem gamblers with those who were classified as non-problem gamblers. Alpha levels were adjusted to control for familywise error rate using Bonferroni correction. A series of multinomial logistic regression analyses were conducted to investigate associations between the five groups of independent variables (demographic, personality, motivational, social, and health) and gambling problems. An additional regression analysis investigated how much of the variance in gambling problems could be explained if all five groups of independent variables were entered into the same model. The criterion in all regression analyses was a categorical variable that grouped the participants in one of three categories: non-gambling, non-problem gambling, and risk-problem gambling, respectively. Non-gambling served as the reference category and thus the analyses addressed the question of whether individual differences on the independent variables affected the likelihood that a person was among the non-problem gamblers or risk-problem gamblers instead of among the non-gamblers. Sample sizes differed slightly between analyses because of missing data on the respective variables. Missing data were removed listwise.

## Results

### Prevalence Rates of Non-problem, Risk, and Problem Gambling

Of the 2,055 17-year-olds who participated, 2,049 could be assigned to one of five gambling categories depending on their sum scores on the PGSI and gambling involvement during the previous month (cf. assignment criteria described in the Method Section). The remaining six participants could not be classified because of missing data.

The vast majority of those who were classified (73.8 %, *n* = 1,513) had not gambled during the previous month (non-gambling). About a fifth (20.3 %, n = 416) had gambled without experiencing any problems (non-problem gambling), 4.1 % (*n* = 84) had gambled with low risk (low risk gambling), 1.5 % (*n* = 31) with moderate risk (moderate risk gambling), and only 0.2 % (*n* = 5) of the participants had experienced gambling problems (problem gambling). If we regard only those who gambled during the previous month, the prevalence rates are 15.7 % for low risk, 5.8 % for moderate risk, and 0.9 % for problem gambling.

Table 1 presents the prevalence data separately for male and female participants. A first visual inspection of the data suggests that males are more likely to have gambled with low or moderate risk than females. The result of a Chi square test confirms that the assignment distribution differs for males and females: *χ* ^2^ (4) = 32.10, *p* < .001, Cramer’s *V* = .125. The standardized residuals (cf. Table 1) show that this effect is mainly due to gender differences in the categories low risk and moderate risk gambling; all four residuals are significant at *p* < .05. The relation between gender and gambling problems will be further investigated in the regression analyses reported below.

**Table 1:** Contingency table gender differences in gambling problems

Gambling category	Male			Female			Total
	Count (%^a^)	Expected count	Standardized residual	Count (%^a^)	Expected count	Standardized residual	
Non-gambling	669 (69.8)	707.4	−1.4	841 (77.4)	802.6	1.4	1,510
Non-problem gambling	205 (21.4)	194.4	0.8	210 (19.3)	220.6	−0.7	415
Low risk gambling	56 (5.8)	39.4	2.7*	28 (2.6)	44.6	−2.5*	84
Moderate risk gambling	24 (2.5)	14.5	2.5*	7 (0.6)	16.5	−2.3*	31
Problem gambling	4 (0.4)	2.3	1.1	1 (0.1)	2.7	−1.0	5
Total	958 (100)			1,087 (100)			2045^b^

* *p* < .05

^a^Percentages within gender (rounded)

^b^Four participants did not answer the gender item and therefore the total *n* here differs by four from the total *n* reported for the prevalence analysis in which gender was not taken into account

The numbers of participants assigned to the risk (low and moderate) and problem gambling categories were too small to carry out meaningful statistical analyses separately for these subgroups. Therefore, we merged the three subgroups into one risk-problem gambling category for the remaining analyses. In the next section, the gambling behavior of those who were assigned to the risk-problem gambling category will be compared with the gambling behavior of those who gambled without reporting problems.

### Gambling Behavior of Problem and Non-problem Gamblers

Those in the risk-problem gambling category played significantly more than those who gambled without reporting problems,^2^ days played *M* = 2.47, *SD* = 5.09 versus *M* = 0.86, *SD* = 1.76, *t* (114.40) = 3.21, *p* < .01. In addition, risk and problem gamblers also lost significantly more money than non-problem gamblers, NOK lost previous month *M* = 79.32, *SD* = 154.89 versus *M* = 22.79, *SD* = 66.15, *t*(131.10) = 3.88, *p* < .001. However, it should be noted that because of missing data the statistics for both variables, days played and NOK lost, are based upon subsamples of participants. Out of 536 participants who had gambled, *n* = 435 reported how many days they had gambled and *n* = 517 reported how much money they had lost in the previous month.

Preferences for gambling options also differed between the non-problem and risk-problem groups. Table 2 lists the eight gambling options that were played by at least 4 % of the participants in at least one of the two groups of gamblers; the shares of participants who played the remaining eight options varied between 0 and 3.3 % (per group).

**Table 2:** Preferences for gambling options per group: numbers and percentages of users (top-8)

Gambling option	Risk-problem gambling (*N* = 120)		Non-problem gambling (*N* = 416)		
	*n*	%	*n*	%	
Scratchcard	58	48.3	295	70.9	*χ* ^2^ (1) = 21.12, *p* < .001, Cramer’s *V* = .198
Gambling on the internet (e.g., betting, poker, casino games)	32	26.7	33	7.9	*χ* ^2^ (1) = 30.67, *p* < .001, Cramer’s *V* = .239
Gambling in closed game clubs/groups (e.g., poker)	29	24.2	24	5.8	*χ* ^2^ (1) = 35.38, *p* < .001, Cramer’s *V* = .257
Odds games	18	15	7	1.7	*χ* ^2^ (1) = 37.15, *p* < .001, Cramer’s *V* = .263
Tipping	16	13.3	21	5	*χ* ^2^ (1) = 9.95, *p* = .002, Cramer’s *V* = .136
Gambling machines	9	7.5	10	2.4	*χ* ^2^ (1) = 7.07, *p* = .008, Cramer’s *V* = .115 Fisher’s Exact Test *p* = .02
Bingo	8	6.7	18	4.3	*χ* ^2^ (1) = 1.11, *p* = .293, Cramer’s *V* = .045
Stock market	5	4.2	9	2.2	*χ* ^2^ (1) = 1.47, *p* = .225, Cramer’s *V* = .052 Fisher’s Exact Test *p* = .325

Bonferroni corrected alpha level of *p* < .006 (i.e., .05 divided by 8). Fisher’s Exact Test is reported for gambling machines and stock market because in both cases one of the expected frequencies in the contingency tables was smaller than 5

The percentages of users show that scratchcards were the most preferred gambling option in both groups. However, the share of those who played scratchcards was larger in the non-problem gambling than in the risk-problem gambling group. Non-problem gamblers mainly used scratchcards whereas the preferences of risk-problem gamblers were more diverse. For example, in both groups the second and third most prominent options were gambling on the Internet (e.g., betting, poker, casino games), and gambling in closed game clubs/groups (e.g., poker). However, the proportion of those who used these options were only 7.9 and 5.8 % (respectively) in the non-problem gambling group but 26.7 and 24.2 % in the risk-problem gambling group. What is more, playing odds games and sports betting (tipping) were more prominent among risk-problem gamblers than among non-problem gamblers.

### Covariates of Gambling Problems

#### Demographic Variables

The results of the multinomial logistic regression analyses are provided in Table 3. In the first analysis, the demographic variables gender, living situation, place of birth, and ethnicity entered as independent variables into the model (Nagelkerke *R* ^2^ = .026).

**Table 3:** Multinomial logistic regressions of gambling problems (reference category: non-gambling, OR = 1)

Independent variables	Non-problem gambling		Risk-problem gambling	
	*B*	OR (95 % CI)	*B*	OR (95 % CI)
Demographic^a^				
Gender (male)	.199^†^	1.22 (0.98–1.52)	1.09**	2.98 (1.98–4.49)
Living situation (lives not with both parents)	.113	1.12 (0.89–1.40)	.283	1.33 (0.90–1.95)
Place of birth (Norway)	.187	1.21 (0.71–2.06)	−.214	0.81 (0.38–1.72)
Ethnicity (Norwegian)	.179	1.20 (0.70–2.04)	−.431	.631 (0.30–1.31)
Personality^b^				
Extraversion	.024	1.02 (0.88–1.20)	−.035	0.97 (0.74–1.27)
Agreeableness	−.199*	0.82 (0.68–.984)	−.376*	0.69 (0.51–0.92)
Conscientiousness	−.088	0.92 (0.78–1.08)	.143	1.15 (0.87–1.53)
Neuroticism	−.139^†^	0.87 (0.75–1.02)	.058	1.06 (0.81–1.38)
Intellect/imagination	−.213*	0.81 (0.66–1.00)	−.328^†^	0.72 (0.50–1.03)
Impulsivity	.068**	1.07 (1.02–1.12)	.176**	1.19 (1.11–1.29)
Sensation seeking	.297	1.35 (0.94–1.92)	1.36**	3.89 (2.08–7.29)
Motivational^c^				
Attitudes toward gambling	.032**	1.03 (1.02–1.05)	.060**	1.06 (1.03–1.09)
Future gambling intentions	.235**	1.27 (1.11–1.45)	.667**	1.95 (1.62–2.34)
Knowledge	.086	1.09 (0.97–1.22)	.475**	1.61 (1.28–2.01)
Social^d^				
Parental monitoring	−.133	0.88 (0.73–1.05)	−.676**	0.51 (0.38–0.67)
Father’s education (lower secondary)	.404*	1.50 (1.05–2.14)	.632*	1.88 (1.06–3.34)
Mother’s education (lower secondary)	−.088	0.92 (0.59–1.42)	−.436	0.65 (0.29–1.42)
Family cohesion	.059	1.06 (0.84–1.34)	.171	1.19 (0.80–1.77)
Family gambling history				
Gambled in lifetime	.169	1.18 (0.82–1.72)	−.189	0.83 (0.41–1.67)
Gambled once a month	−.163	0.85 (0.48–1.50)	.175	1.19 (0.45–3.16)
Gambled once a week	−.652^†^	0.52 (0.27–1.01)	−.941^†^	0.39 (0.13–1.17)
Pathological gambling	1.48*	4.39 (1.29–14.89)	3.09**	21.95 (4.37–110.26)
Family/friends’ approval	.417**	1.52 (1.27–1.82)	1.21**	3.35 (2.44–4.59)
Health^e^				
HADS anxiety	−.006	0.99 (0.96–1.03)	.028	1.03 (0.97–1.09)
HADS depression	.012	1.01 (0.97–1.06)	.117**	1.12 (1.05–1.21)
Loneliness	−.016	0.98 (0.95–1.02)	−.047	.954 (0.90–1.01)

*OR* odds ratio, *CI* confidence interval

^†^ *p* < .10; * *p* < .05; ** *p* < .01

^a^ *R* ^2^ = .026 (Nagelkerke); Model *χ* ^2^ (8) = 39.26, *p* < .001

^b^ *R* ^2^ = .062 (Nagelkerke); Model *χ* ^2^ (14) = 91.13, *p* < .001

^c^ *R* ^2^ = .104 (Nagelkerke); Model *χ* ^2^ (6) = 162.71, *p* < .001

^d^ *R* ^2^ = .097 (Nagelkerke); Model *χ* ^2^ (18) = 147.70, *p* < .001

^e^ *R* ^2^ = .011 (Nagelkerke); Model *χ* ^2^ (6) = 16.01, *p* < .05

Gender was the only significant independent variable: Being male increased the chance of being among the risk-problem gamblers compared to being among the non-gambler subgroups (OR = 2.98). This finding mirrors the gender effect reported in the section on prevalence rates.

#### Personality Variables

Next, a regression model in which the personality variables served as independent variables was computed (Nagelkerke *R* ^2^ = .062; Table 3). Impulsivity and sensation seeking and two of the Big five personality domain traits (i.e., Agreeableness and Intellect/imagination) were found to be significant. More precisely, scoring *lower* on Agreeableness (OR = 0.82) and Intellect/imagination (OR = 0.81) and scoring *higher* on impulsivity (OR = 1.07) increased a participant’s chance of being among the non-problem gamblers compared to the non-gamblers. The chance of being among the risk-problem gamblers compared to the non-gamblers increased with lower Agreeableness (OR = 0.69) and with higher impulsivity (OR = 1.19) and sensation seeking scores (OR = 3.89). Overall, sensation seeking was most strongly associated with risk- and problem gambling.

#### Motivational Variables

The third regression model included the motivational variables as independent variables (Nagelkerke *R* ^2^ = .104; Table 3). Attitudes and future gambling intentions were significantly and positively related to non-problem and risk-problem gambling. In other words, having more positive (or less negative^3^) attitudes toward gambling and having stronger intentions to gamble in the future increased a participant’s chance of being among those who gamble (ORs = 1.03 and 1.27, respectively) and those who gamble with risk and problems (ORs = 1.06 and 1.95, respectively) instead of being among those who did not gamble at all. In addition, gambling-related knowledge was positively associated with risk-problem gambling (OR = 1.61). Thus, feeling more knowledgeable about gambling options puts one at higher risk of being among those who gamble with risk and who have gambling problems. Overall, future gambling intentions was most strongly associated with non-problem and risk-problem gambling among the motivational variables.

#### Social Variables

In a fourth regression model, the social variables served as independent variables (Nagelkerke *R* ^2^ = .097; Table 3). The results show that family and friends’ approval of gambling and having more relatives and/or friends with a history of pathological gambling involvement increase one’s chance of being among non-problem (ORs = 1.52 and 4.39, respectively) and risk-problem gamblers (ORs = 3.35 and 21.95, respectively) compared to being among the non-gamblers. In addition, those who have a father with lower secondary education (highest level of education) are more likely to be among the non-problem (OR = 1.50) and risk-problem gamblers (OR = 1.88). Family monitoring was related to risk-problem gambling only (OR = 0.51). Perhaps not surprisingly, those who indicated that they are less strictly monitored by their parents are more likely to be among the risk-problem gamblers. Overall, having relatives and/or friends with a history of pathological gambling involvement was most strongly associated with non-problem and risk-problem gambling among the social variables.

#### Health Variables

A fifth regression model included the health variables as independent variables (Nagelkerke *R* ^2^ = .011; Table 3). Only the depression subscale of the HADS was significant. Those who score higher on depression are more likely to be among the risk-problem gamblers than among the non-gamblers (OR = 1.12).

The Nagelkerke *R* ^2^ values of the five regression models reveal that the model with the motivational variables distinguishes best between non-gamblers, non-problem gamblers, and risk- and problem gamblers. The model with the social variables explains somewhat less of the observed variance in gambling, followed by the models with the personality, demographic, and health variables.

#### Consolidated Model

Finally, another multinomial regression analysis was performed to address the question of how well all of the above independent variables together explained gambling [*χ* ^2^ (52) = 243.91, *p* < .001, Nagelkerke *R* ^2^ = .180]. Some of the associations that were found between the independent variables and gambling became non-significant in this comprehensive model. Significant odds of non-problem gambling compared to non-gambling were found for (a) impulsivity (OR = 1.06), (b) Intellect/imagination (OR = 0.77), (c) attitudes toward gambling (OR = 1.03), (d) future gambling intentions (OR = 1.21), (e) having more relatives and/or friends with a history of pathological gambling involvement (OR = 5.90), and (f) highest education of father (OR = 1.49). Comparing risk-problem gambling with non-gambling significant odds were found for (a) gambling-related knowledge (OR = 1.55), (b) future gambling intentions (OR = 1.86), (c) family monitoring (OR = 0.59), (d) family and friends’ approval of gambling (OR = 1.67), (e) having more relatives and/or friends with a history of pathological gambling involvement (OR = 19.82), (f) gender (OR = 1.88), and (g) highest education of father (OR = 2.06). All of these associations were in the same directions as in the respective models in Table 3.

## Discussion

In the presented study, we investigated the prevalence rates of gambling and risk- and problem gambling in a representative sample of 17-year-olds in Norway. In addition, we explored the importance of demographic, personality, motivational, social, and health variables for explaining variance in non-problem gambling and risk- and problem gambling.

The prevalence rates of risk and problem gambling found in our study were very low but similar to those reported in previous Australian (Delfabbro et al. 2013) and Swedish studies (Statens folkhälsoinstitut 2010) that used the PGSI in samples of adolescents and young adults. Studies that used different instruments to measure gambling problems in adolescent samples, for example, the DSM IV J (Fisher 1992) or the SOGS RA (Winters et al. 1993), reported higher prevalence rates (Delfabbro et al. 2009; Chalmers and Willoughby 2006). One of the reasons for the lower prevalence rates found in our study may be that the PGSI is a more conservative measure of problem gambling than other available measures when used in non-clinical samples (Jackson et al. 2010). Another possible reason for the low prevalence rates found in our study is that in Norway gambling is highly regulated, and thus adolescents have restricted access to gambling options. The age limit for gambling is 18 years in Norway but some adolescents may find ways to get around this regulation, for example, by using the player account of an adult friend or relative or by gambling in informal settings, such as poker clubs. Other studies conducted in Norway showed that comparatively small proportions of adolescents gamble and experience gambling-related problems. For example, a recent study that used the SOGS-RA found that 1 % of 12–18 year-old Norwegians were problem gamblers and 3.5 % were at-risk gamblers (Frøyland et al. 2010).

In line with previous studies on adolescent gambling, male participants in our sample were more likely than female participants to be at risk for developing gambling problems. A possible explanation for this finding is that, during adolescence, boys are more prone to engage in risk behaviors than girls (Gullone et al. 2000). Apart from gender, no other demographic variables were associated with non-problem and risk- and problem gambling.

As for the relative importance of the covariates, we found that the model with the motivational variables best explained whether participants belonged to the non-gambling, non-problem gambling, or risk-problem gambling group. A possible explanation of this finding is that the vast majority of 17-year-olds in Norway who gamble do not experience serious problems (i.e., symptoms of addiction) related to their gambling. Most participants who were assigned to the group of risk-problem gamblers in our study were actually low or moderate risk gamblers and for these individuals decisions to gamble may be more active and deliberate than for individuals who experience more severe symptoms of gambling addiction. If gambling behavior is under volitional control, then theories to explain deliberate decisions and behavior, such as the theory of planned behavior (Ajzen 1991), may be well-suited for explaining gambling. The theory of planned behavior postulates that a person’s intentions to carry out a behavior are the main determinant of whether the person will actually carry out the behavior. Behavior intentions are determined by a person’s attitudes toward the behavior, perceived social norms, and behavior control (sometimes referred to as self-efficacy). These assumptions are in line with our findings that the motivational variables turned out to be important covariates of gambling and that intentions were a stronger covariate of gambling than attitudes and knowledge (i.e., a component of behavior control). In addition, two of the social variables that were important covariates of gambling in our study may represent descriptive social norms (having relatives and/or friends with a history of pathological gambling involvement) and injunctive social norms (family/friends’ approval of gambling). Previous studies also found that the components of the theory of planned behavior are important covariates of adolescent gambling, for example an early study by Moore and Ohtsuka (1997).

Two other social variables were important covariates in our study: parental monitoring was negatively associated with risk-problem gambling and having a father with lower secondary education was positively associated with non-problem and risk-problem gambling. Adolescents who engage in risk gambling may have to hide this behavior from their parents and this should be easier for those whose parents have a less strict monitoring style. However, those who engage in occasional non-problematic gambling may even be supported by their parents (e.g., getting a lottery ticket as a birthday gift). A possible explanation for the finding that father’s but not mother’s level of education was associated with adolescent gambling is that fathers with lower education may be more likely to engage in risk behaviors (Hampton et al. 2013) and act as strong role model for risk behaviors.

Personality variables were moderately important for explaining adolescent gambling. One of the Big five personality domain traits, Agreeableness, was negatively related to both non-problem and risk-problem gambling; this association has also been found among adult gamblers (Miller et al. 2013). Andreassen et al. (2013) suggested that since addictions often lead to interpersonal conflicts (Wiebe et al. 2000) agreeableness may be a protective factor against addictions as people who score high on this personality trait typically emphasize living in harmony with their surroundings.

This study is among the first to investigate the Big five personality domain traits in connection with adolescent gambling, and our findings indicate that three of the domain traits that have been repeatedly shown to be associated with problem gambling in adult samples, namely Conscientiousness, Neuroticism, and Intellect/imagination (sometimes: Openness) (e.g., Myrseth et al. 2009; Kaare et al. 2009; Miller et al. 2013), have less explanatory power for adolescent gambling. More research is needed to validate this assumption. Additional variance in adolescent gambling was explained by impulsivity (positive association with non-problem and risk-problem gambling) and sensation seeking (positive association with risk-problem gambling). These findings are in line with previous studies (see Introduction Section) and theoretical assumptions, for example, the view that people may engage in excessive and problematic gambling because of difficulties with impulse control (Reuter et al. 2005).

Health variables explained least of the variance in adolescent gambling, and the HADS subscale depression was the only significant covariate (weak positive association with risk-problem gambling) among the health variables. It is often assumed, that excessive gambling can lead to health problems and that individuals with health problems, such as depression or anxiety, may turn to gambling in an attempt to alleviate or get distracted from negative health symptoms (see Introduction Section). The fact that, in our study, prevalence rates of risk- and problem gambling were very low may explain why health variables were less important covariates. Because only few participants were problem gamblers, it is likely that the share of Norwegian 17-year-olds who experience gambling-related health problems is very small. Those adolescents who suffer from health problems may seek alleviation and distraction in other activities than gambling, for example because gambling is not as easily accessible for adolescents in Norway.

We see three limitations of our study that should be considered in future research. Due to the correlational character of the data nothing can be concluded with regard to directionality concerning the associations between gambling and the other variables of interest. The fact that all data were based on self-reports render them vulnerable for social desirability bias (Adams et al. 1999; Miller et al. 2008) and common method bias (Podsakoff et al. 2003). Future studies may solve these issues by including comparative data (McComb and Sabiston 2010), such as parent ratings of the adolescent gambling activities, and by employing longitudinal designs. In addition, direct interviews with adolescents may increase measurement validity. Monetary losses from gambling were measured without further probing if participants had lost money that they won from gambling. Consequently, some may have reported losses from wallet only whereas others may have included losses of “house money” in their answers. To solve this issue, it should be specified in the instructions whether gains from gambling are to be reported or not.

Despite these limitations some assets of the present study deserve mention. The study is one of very few that investigates the exploratory power of a wide range of variables that may be important for better understanding adolescent gambling. The national representativeness and the high response rate should also be emphasized as strengths of the study.

## Footnotes

1 The sample includes responses to the survey that we received before April 1st 2013.

2 Note that some participants who had gambled during the previous month provided a value of less than 1 on the variable *days played*; perhaps, these participants had in mind that they had played less than a full day.

3 A closer inspection of the attitude variable revealed that, on average, participants had a slightly negative attitude toward gambling (mean sum score of *M* = 37.74, *SD* = 7.48; a sum score of 42 would represent a neutral attitude and a sum score greater than 42 a positive attitude).
